# Antenatal Clinic and Stop Smoking Services Staff Views on “Opt-Out” Referrals for Smoking Cessation in Pregnancy: A Framework Analysis

**DOI:** 10.3390/ijerph13101004

**Published:** 2016-10-12

**Authors:** Katarzyna Anna Campbell, Katharine Anna Bowker, Felix Naughton, Melanie Sloan, Sue Cooper, Tim Coleman

**Affiliations:** 1 University of Nottingham, Division of Primary Care, Room 1406, Tower Building, University Park, Nottingham NG7 2RD, UK; katharine.bowker@nottingham.ac.uk (K.A.B.); sue.cooper@nottingham.ac.uk (S.C.); tim.coleman@nottingham.ac.uk (T.C.); 2 Behavioural Science Group, Institute of Public Health, University of Cambridge, Cambridge CB2 0SR, UK; fmen2@medschl.cam.ac.uk (F.N.); mas229@medschl.cam.ac.uk (M.S.)

**Keywords:** “opt-out” referrals, smoking cessation, pregnancy, health support workers, stop smoking services

## Abstract

**Introduction:** UK guidance recommends routine exhaled carbon monoxide (CO) screening for pregnant women and “opt-out” referrals to stop smoking services (SSS) of those with CO ≥ 4 ppm. We explored staff views on this referral pathway when implemented in one UK hospital Trust. **Methods:** Seventeen semi-structured interviews with staff involved in the implementation of the new referral pathway: six antenatal clinic staff (before and after implementation); five SSS staff (after). Data were analyzed using framework analysis. **Results:** Two themes were identified: (1) views on implementation of the pathway and (2) impact of the pathway on the women. Generally, staff felt that following training, referrals were less arduous to implement and better received than expected. The majority believed this pathway helped engage women motivated to quit and offered a unique chance to impart smoking cessation knowledge to hard-to-reach women, who might not otherwise contact SSS. An unexpected issue arose during implementation—dealing with non-smokers with high CO readings. **Conclusions:** According to staff, the “opt-out” referral pathway is an acceptable addition to routine antenatal care. It can help engage hard-to-reach women and educate them about the dangers of smoking in pregnancy. Incorporating advice on dealing with non-smokers with high CO into routine staff training could help future implementations.

## 1. Introduction

Smoking in pregnancy is associated with adverse pregnancy and birth outcomes [1]. Supporting pregnant women to stop smoking is therefore important to protect the mother and child from harm. Evidence has shown that many pregnant smokers do not disclose their smoking status to healthcare professionals [2,3], but engaging them with healthcare services is necessary to provide cessation support, and improves chances of quitting during pregnancy [4]. UK Stop Smoking Services (SSS) provide specialist behavioral and pharmacological support to smokers who want to quit; these services are free of charge at the point of use under the National Health Service (NHS) [5]. The SSS were found to be effective in improving quit rates in smokers who want to stop [6]. In the UK, the National Institute for Health and Care Excellence (NICE) guidelines for smoking cessation in pregnancy recommend an integrated “opt-out” referral pathway from antenatal care to the SSS [7]. This involves assessing all pregnant women attending routine antenatal care for smoking in early pregnancy using systematic exhaled carbon monoxide (CO) screening and referring all with CO ≥ 4 ppm, unless they decline. “Opt-out” pathways have been implemented in order to engage women into smoking cessation services, although few studies to date have investigated the evidence for increased effectiveness [8,9,10].

The attitudes of antenatal staff towards smoking and cessation in pregnancy are considered important factors in determining how smoking is discussed and incorporated into antenatal care [11,12,13]. Some midwives do not discuss smoking because they fear that raising this issue will distress women and thereby jeopardize the creation of a positive relationship [12,13]. Offering smoking cessation support routinely, however, may improve staff’s confidence to discuss smoking with pregnant women [11]. The healthcare environment can further influence how midwives approach the subject, with factors such as lack of time, knowledge, training and other competing tasks identified as potential barriers to discussing smoking in pregnancy [14]. Little is known about what can be done to target the barriers experienced by antenatal staff to improve their attitudes toward “opt-out” referral pathways.

Health support workers (HSWs) are an increasingly common part of the maternity workforce. HSWs work under the supervision of a midwife, and assist in providing care for pregnant and postnatal women [15]. HSWs take responsibility for basic clinical tasks, such as taking blood samples and blood pressure, as well as offering comfort and emotional support to patients. Survey data suggests that, compared to midwives, HSWs are more likely to ask women about smoking, perform CO tests, and refer to SSS when asked to do so as part of their role [8]. While some studies investigated the views of midwives on introducing systematic identification of smokers and routine referrals for smoking cessation in pregnancy [13,16], little is known about the views of HSWs on this subject. 

The introduction of “opt-out” referrals was found to generate a significant increase in referrals to SSS [9,10]. This could potentially have an impact on the professional role and workload of SSS advisers; however, to our knowledge, there are currently no studies investigating the views of SSS staff on introducing the “opt-out” pathway. Understanding the attitudes of all staff involved in an “opt-out” referral pathway could help to identify barriers and facilitators related to implementation and optimization. The aim of this study was therefore to assess HSWs and SSS staff views and attitudes towards routine CO screening and “opt-out” referrals introduced in one NHS Hospital Trust in the East Midlands, UK [10]. This study was a service development evaluation. Furthermore, we interviewed HSWs before and after they underwent comprehensive tailored training and had a chance to practice the pathway for six months, in order to assess how (if at all) their attitudes towards “opt-out” referrals in pregnancy changed with time, practice, and training.

## 2. Materials and Methods

### 2.1. Design

In May 2013, an integrated “opt-out” referral pathway was introduced within one UK NHS Hospital Trust. All five maternity health support workers (HSWs) employed by the trust, their line manager, and a midwife were responsible for implementation and daily running of the pathway within two antenatal clinics in the Trust. Prior to implementation, they were provided with comprehensive evidence-based training from the National Centre for Smoking Cessation Training (NCSCT) [17]. This training aimed to address the concerns about the pathway that staff raised in the first set of interviews described in this paper. Antenatal clinic staff followed an “opt-out” referral pathway protocol described in more detail elsewhere [10]. According to the protocol, all pregnant women attending their first antenatal scan (8–14 weeks gestation) were asked to provide an exhaled carbon monoxide (CO) sample, and those with CO ≥ 4 ppm were referred to the local SSS, unless they specifically declined. This cut-off point is thought to be optimal for identifying pregnant smokers [9,18]. The antenatal clinic manager’s role was to support staff and oversee the pathway. One of the five HSW was appointed as an additional part-time member of the team for six months to help with the potential increase in workload at the start of the project. The pathway was implemented in addition to the Trust’s standard care, where women were asked about their smoking status without CO validation by a midwife during their booking appointment at around 8–12 weeks gestation, with those who wanted support being referred to SSS (“opt-in” referrals). All “opt-out” referrals were sent electronically to the local SSS, where the advisers attempted to telephone each woman twice, and if unsuccessful, they sent a letter inviting them to call for support. Women who engaged with the service received up to 12 weeks of behavioral support [19] and nicotine replacement therapy when required. 

Similar numbers of women attended the dating scan appointment in the six-month period immediately after the implementation of the “opt-out” referral pathway (May–October 2013; *N* = 2011), compared to a time-matched period before the implementation (May–October 2012; *N* = 1975). While there was no CO testing offered to women at this appointment before the “opt-out” pathway was introduced, after the implementation, 80% of women attending scans were offered the CO test (*n* = 1610), and 25% had a level of CO ≥ 4 ppm (*n* = 506). As a result of the introduction of the “opt-out” referral pathway, the number of pregnancy referrals sent to SSS increased by 45% (from 290 in the period before implementation to 421 after implementation). Of those who were referred to SSS after implementation, around 30% engaged/set a quit date with the SSS (compared to 20% before), and the four-week cessation rates doubled (from 46 before to 93 after implementation). More information about the design and a detailed outcome evaluation of this service development was published elsewhere [10]. 

### 2.2. Procedure and Participants

**Figure 1:**
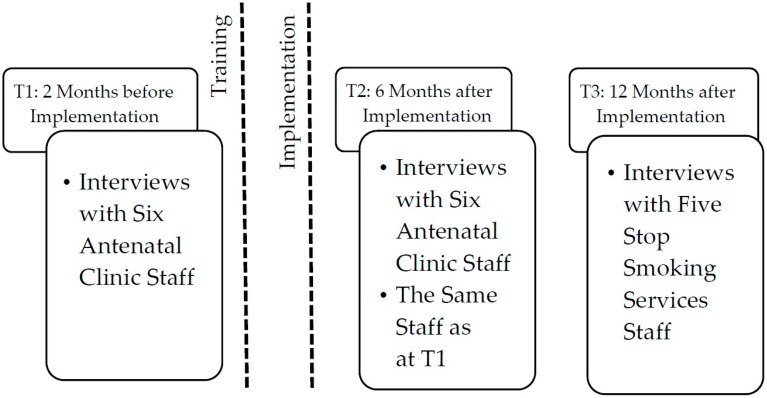
Interview timeline.

**Figure 2:**
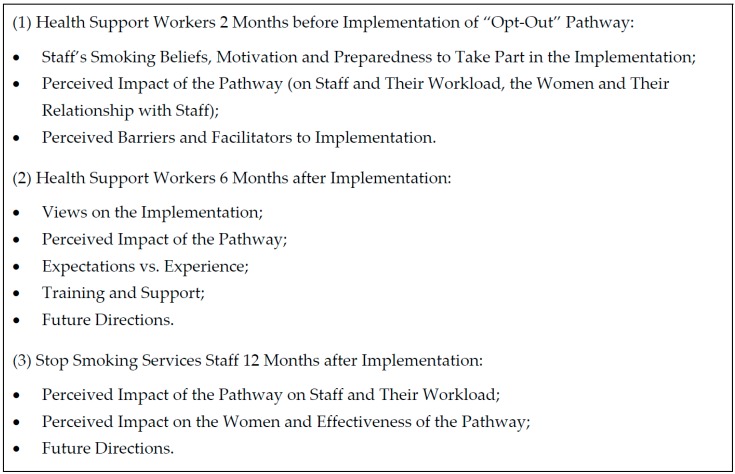
Interview topics.

All HSWs and SSS staff directly involved in the implementation of the “opt-out” referral pathway were eligible to take part in this study. All eleven staff members involved in the pathway were invited to take part in the interviews (at the antenatal clinic: all five HSWs and their manager, henceforward referred to as HSWs for confidentiality; at the SSS: all five SSS staff involved in the pathway, including two advisers, two administrators, and their manager). Staff were approached by the researchers with the managers’ permission, and they all agreed to take part in the interviews. As a result, all HSWs and all SSS staff involved in this project were interviewed, and 17 face-to-face semi-structured interviews were conducted at three time-points. Details of the interview timeline are presented in Figure 1. Interview topic guides are presented in Figure 2. All staff were female, the time in their current role varied between 1 and 33 years, and one reported to be a current smoker. Interviews were conducted by two authors (KAB, female researcher with midwifery background; and KAC, non-clinical female researcher) in a private room within the interviewees’ places of work. Both researchers were involved in the implementation of the “opt-out” pathway, and had an established relationship with participants as well as past experience in conducting qualitative research. The interviews lasted between 15 and 40 min, were audio-recorded and transcribed verbatim.

### 2.3. Ethical Considerations

Routine antenatal care was changed by the Trust for all pregnant women attending their antenatal scan appointment; therefore, this project was categorized as a “service development evaluation” rather than research, by the National Research Ethics Service. In the UK, unlike research, “service development evaluations” do not require ethical approval. We contacted the NHS Trust directly and obtained a written permission to carry out the evaluation. All participants received an information sheet containing a brief description of the project/aims of the interviews, their rights as participants, and written informed consent was collected prior to the interviews.

### 2.4. Analysis

Framework analysis was employed to manage, summarize, and interpret the data [20]. When using framework analysis, data from individual participants are mapped onto a framework matrix. This process offers a clearer understanding of the views, opinions, and experiences of each participant, which is particularly useful when distinct groups of participants contribute to the data. As we interviewed HSWs and SSS staff, framework analysis was deemed appropriate. Because little is known about the views and experiences of these health professionals on the implementation of “opt-out” referrals in pregnancy, the data were analyzed inductively.

The two researchers who conducted the interviews undertook the analysis, using NVivo (Version 10, QRS International Pty Ltd., Melbourne, Australia) to organize data. Seven stages of framework analysis described by Gale and colleagues [20] were followed: (1) interview recordings were transcribed verbatim; (2) the researchers thoroughly familiarized themselves with the dataset; (3) the researchers independently assigned open codes to the transcripts; (4) the researchers discussed the coding and agreed on a final working analytical framework, and each category and subcategory of coding was labelled and defined; (5) the framework was applied to the entire data; (6) data from each transcript were chartered onto a framework matrix; (7) the data were interpreted using the framework matrix and initial notes from the process. 

## 3. Results

The responses of the participants were categorized into two broad themes: (1) views on implementation of the pathway, including the impact it had on their professional roles, their reflections on their own confidence to administer the pathway, and their ability to engage the pregnant women; (2) impact of the pathway on the women, both smokers and self-reported non-smokers.

### 3.1. Views on Implementation of the Pathway

#### 3.1.1. Perceived Impact on Professional Roles

Before pathway implementation, the HSWs felt they were busy in their current role, and already managed multiple tasks. They were concerned that the time necessary to engage in the “opt-out” pathway may affect their other duties. Not having enough staff to share the workload, and the ongoing increase in responsibilities was also a concern:

“Just the fact that they keep giving us more jobs here. We get more and more jobs. We haven’t got enough time to actually do all the jobs.”

(p. 5, HSW, Before)

After implementation, the HSWs found that introduction of the pathway had less impact on their time than anticipated. They reported that they initially felt overwhelmed by the changes, but quickly became confident in the new tasks. They also indicated that having an extra staff member helped lessen the impact on their workload.

SSS staff also reported that the volume of new referrals initially had a significant impact on their workload, but this became more manageable with time, as new procedures were introduced:

“To begin with there was a lot more (referrals) than there is now. It gave us quite a big workload, but we’ve worked through it, there’s SOPs (standard operating procedures) on what we should do and how it’s to be done and as soon as that was put in to place it was a hell of a lot easier to do.”

(p. 10, SSS, After)

SSS staff also noted that women referred via the “opt-out” pathway had less understanding of cessation services, which made “opt-out” referrals more labor intensive. They highlighted that while their “usual” clients expect their call, women with “opt-out” referrals were often unsure who was calling them and why; therefore, they required a more detailed introduction and explanation before they could start discussing available support:

“I think the difficulty was that with many of our other referrals people are choosing to refer themselves and they’re fully aware of exactly what they’re being referred in to. That was the difficulty with these women. So I guess in that sense they didn’t always get the same as that other referral, because they didn’t know why we were calling them. And that’s why sometimes it took a lot more work.”

(p. 7, SSS, After)

SSS staff also felt that many women with “opt-out” referrals did not attend appointments, resulting in wasted staff time. They suggested that these women may never have had the intention or motivation to quit and agreed to an appointment just to end the telephone conversation with the advisor. Conversely, this perception might have been related to the significant increase in the overall number of referrals, as effectively more women declined support in the period after implementation (300 out of 421 referred) than were referred in the period before (290).

#### 3.1.2. Confidence in Administering the Pathway

Smoking cessation was a new subject to embrace for most of the HSWs. Before implementation, some felt anxious about broaching the subject, as they did not want to appear judgmental, alienate women who smoke, or unjustly “accuse” those who don’t, and thus compromise the trust between them and the women:

“I think that’s how they might see it, some of them, that you’re either being patronizing or you’re accusing them of smoking when they don’t, which I’m not going to be doing either, I’m just going to be asking a question for their wellbeing, it’s not my wellbeing, it’s their we’re concerned with.”

(p. 2, HSW, Before)

After implementation, however, the majority of HSWs felt it had little impact on their relationships with the women and that women were generally accepting of the new procedures. 

CO monitoring revealed that some self-reported non-smokers had increased CO levels; the HSWs reported they had not envisaged the high levels of anxiety this could cause to some women. They felt unsure how to handle this, were keen to avoid questions and often felt helpless when dealing with this issue:

“I feel a bit bad because I’ll know my next question from them is ‘well why is there (high CO reading) and what does it mean and what’s going to happen’, and I’ll have to say ‘I’m sorry, look I don’t know!’”

(p. 2, HSW, After)

SSS staff also found the high CO readings in self-reported non-smokers difficult to explain. They described addressing air pollution, second-hand smoking, and faulty gas appliances, but in some cases there appeared to be no explanation. One member of SSS staff described attempts to find additional information, but she felt there wasn’t enough research:

“Yeah it’s a really difficult number (4–7 ppm), because obviously it is triggering a referral, but we just didn’t know why (…) so I tried to find some research about that, but there’s nothing out there. Nobody got back to me. I rang [the CO monitor manufacturer] as well and tried to get information from them as to why we might get false readings.”

(p. 7, SSS, After)

#### 3.1.3. Engaging the Women

Before implementation, some HSWs worried that certain women may be reluctant to accept CO screening, particularly those who may “not want to be found out as smokers” (p. 6, HSW, Before). One interviewee expressed concerns that women may not turn up for appointments because they “might be scared that they’ll be frowned upon” (p. 4, HSW, Before). After implementation, they did not think this was happening, and one interviewee also noted that “telling the women” rather than “asking them” to take the test/referral (which is in line with the “opt-out” nature of the pathway) improved compliance:

“We don’t ask them, we tell them that this is a test we’re going to do. I don’t actually ask, but then when I’ve told them what test we’re going to do, I do say ‘is that alright’ and most of them just say ‘yes’ , but a few have refused.”

(p. 5, HSW, After)

Some of the SSS staff suggested that simply referring smokers to SSS may not have been sufficient to engage the hard-to-reach groups. They felt that a more direct and immediate approach involving a face-to-face appointment with a specialist smoking cessation advisor at the same time as the CO test could help improve women’s motivation to stop smoking and increase their engagement with SSS.

### 3.2. Impact of the “Opt-Out” Referral Pathway on the Women

The interviewees indicated that the “opt-out” pathway resulted in an influx of referrals from three distinct groups of women: self-reported non-smokers with high CO level; smokers who were not ready to quit; and smokers who were ready to make a positive change with regards to smoking. It was apparent that they felt the pathway impacted each of these groups differently. Furthermore, the impact of the pathway on the women was discussed in terms of two types of outcomes—the immediate behavioral effects (such as setting quit dates and making a quit attempt), and potential changes in beliefs and attitudes towards smoking in pregnancy that could impact smoking behaviors in the future. 

#### 3.2.1. Impact on Non-Smokers with CO Levels ≥ 4 ppm

As mentioned previously, high CO levels were distressing to some self-reported non-smokers; nevertheless, SSS staff noted that contacting these women had positive outcomes, as it gave them a unique chance to talk about second-hand smoking, smoke-free homes, and smoking around children. They found it useful, as they felt that non-smokers would not contact the services otherwise, and the explicit knowledge about the dangers of second-hand smoking might impact their attitudes towards smoking indoors and being around smokers.

#### 3.2.2. Impact on Smokers Not Ready to Quit

Even before implementation, HSWs felt that some women—particularly those from hard-to-reach communities with strong normative beliefs about smoking—may be difficult to engage with cessation support, as smoking is an integral part of their lives. They felt these beliefs could negatively impact their willingness to engage with SSS; however, the “opt-out” pathway could be a way to challenge these beliefs:

“I suppose it’s difficult sometimes because there’s women that smoke and have had babies that are perfectly well, so it’s hard to get through to somebody that’s seen that, because they can turn round and say ‘well, my sister’s just had a baby and she smokes and her baby’s absolutely fine, so why should I stop smoking’…But I suppose some people don’t understand the complications as much, so I suppose they should have the options there, that this could happen and that could happen.”

(p. 4, HSW, Before)

The SSS staff thought that overall the “opt-out” pathway might have had less impact on the immediate number of women in this group who quit smoking, but they also felt that the pathway helped raise awareness of the dangers of smoking and hopefully influence future behaviors:

“I think ‘the ‘opt-out’ pathway’ is just something else that contributes to that drip, drip, drip effect…It may be that they continue to smoke for that pregnancy, but for the next pregnancy they think about it and they’ve quit. You know it’s really hard to measure…”

(p. 8, SSS, After)

#### 3.2.3. Impact on Smokers Ready to Make a Positive Change

While some women were not ready to change their smoking habits, SSS staff found that others were ready to accept support. The HSWs suggested that seeing the CO levels helped motivate women to make a positive change to their smoking habits. The SSS staff felt these could be women who would eventually contact the service anyway, but the CO test and the referral gave them the extra incentive they needed, and the call from the SSS offered the “final push”:

“With the CO reading we get a lot of clients who say that they are going to face-to-face appointments because we can see the CO reading and they can see how it’s affecting them internally as well as, you know, externally really…because it kind of is a shocker I think to some people.”

(p. 9, SSS, After)

The “opt-out” pathway also gave SSS staff the chance to make an impact on the lives of those who had already tried to cut down. They took this opportunity to help them understand the benefits of quitting over cutting down and to see cutting down as a process that can help them move towards quitting as the final goal.

## 4. Discussion

This is the first study to explore the views of HSWs and SSS staff involved in an “opt-out” referral pathway in pregnancy. As we interviewed HSWs before and after training and implementation, we have also been able to capture how perceptions of these health professionals change over time, how initial concerns get resolved during implementation, and learn about any unforeseen issues that emerged during implementation. 

Before implementation of the pathway, the HSWs raised concerns with regards to discussing smoking that were similar to those raised by midwives in past research: time constraints and perceived lack of expertise [13,14]. After implementation, however, the participants felt the referrals were less arduous to implement than expected. One possible explanation is that the HSWs received tailored training, and concerns voiced in the first round of interviews were considered when designing training material. During training sessions, they had the opportunity to discuss important issues and learn new skills, such as how to explain the harms of smoking in pregnancy or how to deal with women reluctant to take the CO test. Support from the additional HSW also helped to distribute the workload. SSS staff found this pathway relatively easy to adapt to, once standard operating procedures were in place; however, additional workload was considerable, and appointing an additional SSS staff member could aid future implementations. Another study which explored the views of pregnant women who experienced this pathway [21] also suggested that increasing the number of staff who deal with the additional referrals at SSS is advisable. Several of the women interviewed complained that they did not receive a call from SSS, which indicates that two phone calls and a letter may not be sufficient to get hold of all referred women.

Past research suggested that antenatal staff often felt unprepared to give complex cessation advice and preferred to refer women to the SSS, which was a barrier to them engaging in smoking cessation support for pregnant women [13,14]. Our project required HSWs to refer women after giving only very brief advice on smoking [7,19]. Their acceptance of the pathway could suggest that antenatal staff may be more willing to take on less complex roles in smoking cessation. Furthermore, in past studies, midwives indicated that other antenatal health issues (such as obstetric complications) were of higher priority than smoking [13,14]. This was not raised by HSWs in this study, and may highlight the different responsibilities of midwives and HSWs, suggesting that HSWs may be better placed to deliver an “opt-out” pathway in antenatal settings. Hiscock and colleagues [22] also found HSWs to be more effective in providing smoking cessation advice than qualified nursing staff, and they suggest that although HSWs may have fewer medical qualifications, they have more time to build rapport with the clients and address smoking.

HSWs were initially concerned about offending women by discussing smoking and thus damaging their relationship with the patient. While such concerns are common amongst health professionals [11], most women expect to be asked about smoking during pregnancy [23]. Indeed, after implementation, the HSWs felt women accepted the CO reading/referrals as part of their routine care. This was largely confirmed by the views of the women, who felt that CO screening and, to a somewhat lesser degree, “opt-out” referrals were an acceptable part of routine antenatal care [21]. 

The optimum CO cut-off point for pregnant smokers is 4 ppm. This has been defined for optimum specificity and sensitivity, enabling light smokers to be identified [9]. An unexpected issue arose after implementation of the “opt-out” referral pathway, which involved dealing with self-reported non-smokers with CO ≥ 4 ppm. Some HSWs felt uncomfortable being unable to offer women an explanation for this. Incorporating possible explanations for increased CO in non-smokers into future training could help staff deal with this issue. Increasing the threshold may not be an ideal solution, as the low cut-off point helped identify women exposed to second-hand smoke and gave SSS staff a chance to impart advice on smoke-free homes. 

“Opt-out” referral pathways aim to improve cessation outcomes. SSS staff indicated that some of the women referred via the pathway were ready to make positive changes and appreciated the encouragement. An outcome evaluation of the pathway showed that the numbers of women setting quit dates with the services and reporting short term abstinence doubled in the period after implementation, compared to the time-matched comparison period [10]. Conversely, many women contacted by the SSS staff were not ready to make changes to their smoking behaviors at the time. By having increased contact with the latter group, the SSS felt they could influence attitudes and behavior by educating and raising their awareness about smoking cessation support in hard-to-reach communities. Informing women from these communities about the dangers of smoking in pregnancy may, in the long term, help challenge community normative beliefs about the safety and acceptability of smoking in pregnancy. This could in turn lead to more women considering quitting in current or subsequent pregnancies. Providing information about the SSS support available could also increase self-referrals to SSS for professional support.

We recognize some limitations to the work presented. The interviewers had prior involvement with the participants throughout the project, which may have influenced the responses. However, as the interviewers were not part of the NHS, it may have enabled participants to feel more comfortable to talk about their working environment. Generalizability of findings could also potentially be an issue; we interviewed staff involved in the implementation of an “opt-out” referral pathway in one relatively large NHS hospital Trust serving a disadvantaged neighborhood in the East Midlands, UK, where awareness of SSS is likely to be low and smoking at time of delivery tends to be higher than the national average [24]. Thus, the findings may be most generalizable to maternity hospitals in similar economically disadvantaged areas, where smoking prevalence is high. The fact that all participants were female is also a limitation; however, midwives and HSWs who serve pregnant women tend to be predominantly female [25]. Thus, the sample is representative of the wider population. Finally, the non-randomized design of the main “opt-out” study [10] and the lack of a control group made it impossible to compare the views of staff who did and did not undergo training before implementation of the pathway. Whilst we explored how the HSWs’ views changed from before and after training and implementation, future research could add to this knowledge and help compare the views of HSWs who did and did not receive tailored training before the implementation of an “opt-out” pathway in order to better understand the usefulness of such training. 

This study has several strengths. To our knowledge, this is the first comprehensive attempt to explore the views of all staff involved in the implementation of an “opt-out” pathway, both in the antenatal clinic and SSS. The findings provide an interesting and invaluable insight into a process that according to NICE guidelines [7] should be implemented nationwide. Exploration of the views and attitudes of staff before and after implementation offered a unique insight into barriers towards introducing “opt-out” referrals with CO identification of smokers, and how they change with time and through tailored training and peer support. The current study presents the views of two groups of health professionals who are key to smoking cessation in pregnancy but have received less attention in the literature. While other Trusts may delegate the implementation of the “opt-out” referral pathway to health professionals other than HSWs, many perceived barriers prior to implementation reported by the HSWs in our study are comparable to those reported by midwives in past research [13,14], suggesting that antenatal staff share similar concerns in this matter. However, our findings suggest that HSWs in this Trust have adapted well to the “opt-out” pathway and found it acceptable as part of their routine. Involving this group of health professionals in cessation advice could therefore be adopted by other Trusts, freeing up the time of midwives. Lessons learned from this study could help aid implementation of “opt-out” referral pathways in maternity hospitals throughout the UK.

## 5. Conclusions

Overall, the results of this study showed that despite their initial concerns, staff felt that this way of referring pregnant smokers to SSS was relatively easy to implement and well received by the patients. With tailored training and additional support, both HSWs and SSS staff were able to incorporate the “opt-out” pathway into their daily routines. Participants felt that the pathway helped engage women already prepared to make a positive change and offered a unique chance to improve knowledge and awareness of SSS amongst women from hard-to-reach groups, who would not otherwise engage with the service.

Our findings offer insight which may help refine future implementations of “opt-out” referrals in other hospitals. Future recommendations include more support (i.e., appointing additional staff) to help SSS increase the reach of the services, in particular to increase their capacity to engage women. Extra support and information may be required to assist staff to advise non-smokers with unexplainable high CO readings.
